# Potential Adverse Cardiovascular Effects of Treatment With Fluoxetine and Other Selective Serotonin Reuptake Inhibitors (SSRIs) in Patients With Geriatric Depression: Implications for Atherogenesis and Cerebromicrovascular Dysregulation

**DOI:** 10.3389/fgene.2019.00898

**Published:** 2019-09-20

**Authors:** Zoltan Ungvari, Stefano Tarantini, Andriy Yabluchanskiy, Anna Csiszar

**Affiliations:** ^1^Reynolds Oklahoma Center on Aging, Department of Geriatric Medicine, University of Oklahoma Health Sciences Center, Oklahoma City, OK, United States; ^2^Translational Geroscience Laboratory, Department of Geriatric Medicine, University of Oklahoma Health Sciences Center, Oklahoma City, OK, United States; ^3^Department of Medical Physics and Informatics, Faculty of Medicine and Faculty of Science and Informatics, University of Szeged, Szeged, Hungary; ^4^Department of Public Health, Semmelweis University, Budapest, Hungary

**Keywords:** vascular cognitive impairment, atherosclerosis, senescence, antidepressant, aging

## Abstract

Late life depression is an important public health problem, which associates with increased risk of morbidity and mortality. Selective serotonin reuptake inhibitors (SSRIs), including fluoxetine, are often prescribed to treat geriatric depression. There is increasing evidence that fluoxetine and other SSRIs exert a wide range of cardiovascular side effects. Furthermore, there is evidence that aging may increase plasma level of SSRIs. In this overview, the potential role of side effects of treatment with fluoxetine and other SSRIs in the pathogenesis of age-related cardiovascular diseases, including atherogenesis, cardiac pathologies, and cerebromicrovascular impairment, is discussed.

## Introduction

Major depression occurs in 1% to 3% of older adults and an additional 8% to 16% of elderly patients present with clinically significant depressive symptoms (Blazer, 1989; Reynolds et al., 1993; Cole and Dendukuri, 2003). The prognosis of geriatric depression is poor (Cole and Dendukuri, 2003). Geriatric depression associates with poorer functioning, impairs cognition, increases risk of death from illness, reduces an elderly person’s ability to rehabilitate, increases the perception of poor health and the utilization of medical services, and results in significant increase in health care costs (Cole and Dendukuri, 2003). Despite the clinical and societal importance of geriatric depression, it is estimated that only 10% to 20% of patients are diagnosed and adequately treated. Numerous antidepressant drugs have been tested for the treatment of geriatric depression. Among them, selective serotonin reuptake inhibitor (SSRI) drugs, including the potent SSRI fluoxetine, are widely prescribed to treat depression in geriatric patients.

**Figure 1:**
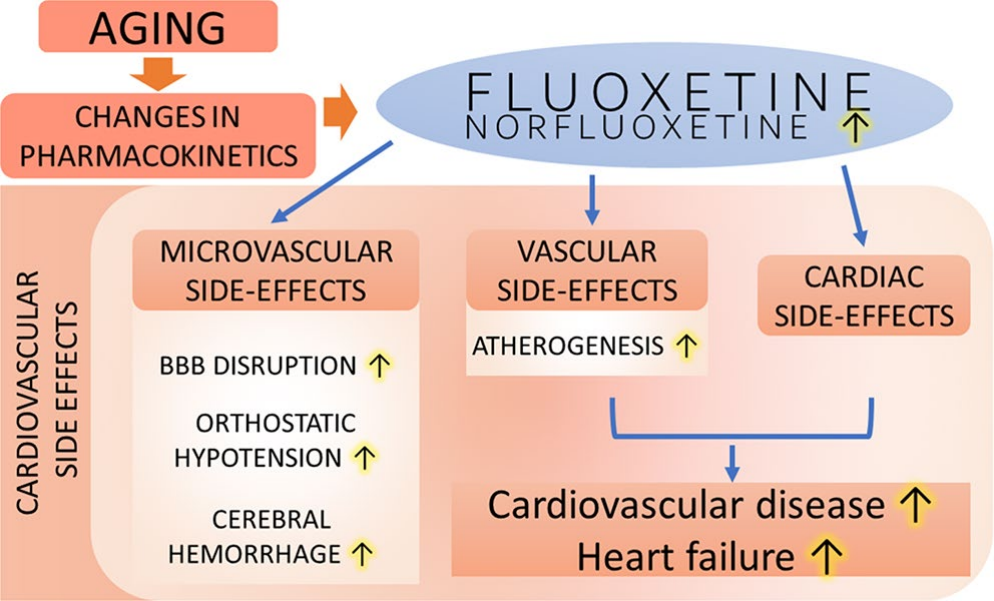
Proposed scheme depicting potential cardiovascular side effects of SSRI (e.g., fluoxetine) treatment in depressed geriatric patients. The model predicts that due to age-related changes in pharmacokinetics plasma concentrations of SSRIs (shown are fluoxetine and its active metabolite, norfluoxetine) increase. Fluoxetine and other SSRIs may inhibit voltage-dependent Ca^2+^ channels in the vascular smooth muscle cells, attenuating myogenic constriction of resistance arterioles, which may promote orthostatic hypotension and/or exacerbate pressure-induced microvascular damage [promoting disruption of the blood-brain barrier (BBB) and exacerbating cerebral hemorrhages]. Cardiac side effects of SSRIs combined with its effect on atherogenesis may promote cardiovascular disease and exacerbate heart failure in geriatric patients.

In this overview, the potential vascular side effects of treatment with fluoxetine and other SSRIs in geriatric patients are considered in terms of both the potential proatherogenic effects of the drug and its ability to impair vascular autoregulatory mechanisms (**Figure 1**).

## Age-Related Changes in Pharmacokinetics of Fluoxetine and Other SSRIs

With aging, absorption, distribution, metabolism, and excretion of drugs frequently change. Older depressed patients treated with tricyclic antidepressants have been reported to be sensitive to drug toxicity resulting from age‐related pharmacokinetic changes and polypharmacy. Despite a large body of evidence documenting the efficacy and safety of fluoxetine (Prozac) and other SSRIs, there are relatively few published data characterizing age-related changes in pharmacokinetics of fluoxetine and other SSRIs in older patients.

Fluoxetine is well absorbed after oral intake, but it is extensively affected by first-pass metabolism in the liver (Altamura et al., 1994). Its active metabolite, norfluoxetine, is formed by demethylation of fluoxetine. The cytochrome P450 enzymes CYP2D6, CYP2C19, CYP2C9, CYP3A4, and CYP3A5 have been implicated in the biotransformation of fluoxetine to norfluoxetine (Margolis et al., 2000; Ring et al., 2001). Despite abundant evidence from preclinical studies demonstrating aging-induced reduction in CYP content, activity, and/or inducibility, human studies have yielded mixed results and failed to demonstrate a consistent decline in CYP in older adults (Kinirons and O’Mahony, 2004). The primary route of elimination of fluoxetine/norfluoxetine is predominantly through oxidative metabolism and conjugation (Gram, 1994). Fluoxetine/norfluoxetine is mainly excreted in urine. The elimination half-life of fluoxetine and norfluoxetine is approximately 1 to 4 days and 7 to 15 days, respectively (Altamura et al., 1994). Fluvoxamine has no active metabolites.

The extent of absorption of other SSRIs, including paroxetine, sertraline, and citalopram, from the gastrointestinal tract is generally good (van Harten, 1993). Following absorption paroxetine is almost completely metabolized in the liver (van Harten, 1993). Sertraline also undergoes extensive metabolism (van Harten, 1993). Known pathways for metabolism of citalopram include N-demethylation, deamination, and N-oxidation to less lipophilic compounds to be excreted in urine (van Harten, 1993) (∼12% of a 40-mg dose of citalopram was reported to have been recovered in urine as citalopram, 12% as the main metabolite demethyl-citalopram, 1.5% as amino metabolite, and 4.3% as conjugated propionic acid metabolite (van Harten, 1993)). While paroxetine, sertraline, and citalopram do have pharmacologically active metabolites, these are not believed to contribute significantly to the overall clinical effect profile of these SSRIs (van Harten, 1993).

The metabolism of paroxetine, citalopram, and sertraline is impaired in older adults. Steady-state plasma concentrations in the elderly are about twice those measured in younger subjects, and the half-life of paroxetine is twice as long in older adults as in younger individuals (van Harten, 1993). The steady-state pharmacokinetics of citalopram are significantly affected by aging. Steady-state citalopram plasma concentrations after a once-daily 20-mg dose in older individuals were reported to be approximately four times higher than in younger subjects (van Harten, 1993). Plasma concentrations of sertraline were also reported to be higher in older subjects than in young individuals (van Harten, 1993). In contrast with its effect on the pharmacokinetics of other antidepressants, initial studies reported no major age-dependent differences in fluoxetine pharmacokinetics (Bergstrom et al., 1983; Altamura et al., 1994). However, in subsequent studies, it was reported that in older adults following oral treatment with fluoxetine the plasma concentration of its active metabolite norfluoxetine reaches higher levels than in younger individuals (Preskorn, 1993; Ferguson and Hill, 2006). The terminal half-life of norfluoxetine was also reported to be longer in patients older than 75 years (Ferguson and Hill, 2006). There appears also a sex difference in pharmacokinetics, as elderly women tend to exhibit higher serum levels of norfluoxetine than men (Ferguson and Hill, 2006). The long half-lives of fluoxetine and norfluoxetine likely promote insidious drug accumulation and manifestation of side effects. Polypharmacy is a common practice in geriatric patients, and potential drug–drug interactions may also affect plasma levels of fluoxetine and norfluoxetine in this patient group.

## Vascular Side Effects of Fluoxetine and Other SSRIs: Potential Pathogenic Role of Impaired Myogenic Constriction of Resistance Arteries

The main mechanism of action of SSRIs, including fluoxetine, depends on their effect to increase the extracellular level of the neurotransmitter serotonin by limiting its reuptake into the presynaptic neurons. This effect results in increased level of serotonin in the synaptic cleft, facilitating its binding to its postsynaptic receptor. However, in the past two decades, preclinical studies demonstrated that fluoxetine, and other SSRIs, also exert significant other cellular effects that are independent of their ability to inhibit serotonin reuptake and involve antagonism of serotonin receptors and inhibition of L-type and T-type voltage-dependent Ca^2+^ channels, as well as other ion channels (Pacher et al., 1998; Pacher et al., 1999a; Ungvari et al., 1999; Deak et al., 2000; Pacher et al., 2000; Ungvari et al., 2000; Terstappen et al., 2003; Traboulsie et al., 2006; Jurkiewicz et al., 2012; Gobin et al., 2015). Importantly, fluoxetine, as well as other SSRIs, was shown to exert significant vascular effects, including inhibition of pressure-induced myogenic constriction of blood vessels (Pacher et al., 1999a; Ungvari et al., 1999; Ungvari et al., 2000) (**Figure 1**). These vascular effects appear to be mediated by inhibition of L-type voltage-dependent Ca^2+^ channels in the vascular smooth muscle cells (Pacher et al., 1999a; Ungvari et al., 1999; Ungvari et al., 2000). These preclinical observations have important clinical relevance. First, impaired pressure-induced myogenic constriction of peripheral resistance arteries has been implicated in the pathogenesis of orthostatic hypotension. Orthostatic hypotension is very common in geriatric patients and associates with significant morbidity and mortality (Gupta and Lipsitz, 2007; Lipsitz, 2017). Adverse consequences of orthostatic hypotension include falls, fracture, functional decline, and myocardial ischemia. The peripheral vascular effects of SSRIs that promote orthostatic hypotension may be exacerbated by its ability to cause bradycardia as well (Pacher et al., 1998; Pacher et al., 1999b).

Second, pressure-induced myogenic constriction of cerebral resistance arteries is critical for normal cerebral autoregulation and protection of the vulnerable distal part of the microcirculatory network from penetration of high pressure (Toth et al., 2017). Recent studies demonstrate that aging impairs myogenic autoregulatory protection in the brain (Toth et al., 2013a; Toth et al., 2013b; Toth et al., 2014; Springo et al., 2015; Toth et al., 2017), potentially exacerbating cerebromicrovascular injury, blood-brain barrier disruption, and neuroinflammation. Importantly, fluoxetine was shown to significantly attenuate pressure-induced myogenic constriction of cerebral arteries (Ungvari et al., 1999). The inhibitory effects of fluoxetine on myogenic constriction of cerebral resistance arteries are likely mediated by its calcium antagonist effects (Ungvari et al., 1999). Indirect evidence suggests that other SSRIs may have similar effects on smooth muscle calcium homeostasis (Pacher et al., 2001; Matchkov et al., 2015). In addition, fluoxetine was shown to inhibit cytochrome P450–mediated metabolism of arachidonic acid and reduce the formation of 20-HETE by over 70%, at least in part, by down-regulating CYP4A proteins (Yuan and Rapoport, 2015). These are important findings as genetic deficiency in the CYP4A1-mediated formation of 20-HETE was shown to contribute to impaired myogenic response in cerebral arteries and impaired autoregulation of cerebral blood flow (Fan et al., 2015; Fan et al., 2016). Further, there is also evidence that administration of drugs that inhibit voltage-dependent Ca^2+^ channels or biosynthesis of 20-HETE abolishes myogenic constriction of cerebral arteries *in vitro* (Toth et al., 2013a; Toth et al., 2013b; Toth et al., 2014; Springo et al., 2015) and/or autoregulation of cerebral blood flow *in vivo* (Pearce and Bevan, 1987). Impairment of myogenic constriction has been linked to increased incidence of intracerebral hemorrhages in aging (Toth et al., 2013b; Toth et al., 2014; Toth et al., 2015b; Tarantini et al., 2017c). In that regard, it is significant that there is epidemiological evidence suggesting that treatment with SSRIs associates with an increased risk of intracerebral hemorrhages (Hackam and Mrkobrada, 2012). There is also evidence that pathological events traditionally described as “transient ischemic attacks” are, in fact, due to cerebral microhemorrhages. Interestingly, there are clinical observations that initiation of treatment with SSRIs can promote these events (Manos and Wechsler, 2004). Thus, it should be determined whether in older individuals higher plasma concentrations of SSRIs, including fluoxetine and norfluoxetine, have an exacerbated inhibitory effect on cerebral autoregulatory protection, potentially promoting cerebromicrovascular injury.

## Vascular Side Effects of Fluoxetine and Other SSRIs: Potential Pathogenic Role in Atherogenesis

During the past two decades, increasing evidence has been published, suggesting that treatment of older adults with antidepressants may exert adverse effects on cardiovascular disease outcomes (Cohen et al., 2000; Schlienger et al., 2004; Tata et al., 2005). Previous studies reported that treatment with non-SSRI tricyclic antidepressants results in an increased risk of atherosclerotic vascular disease in older adults (Cohen et al., 2000). Importantly, epidemiological studies analyzing participants from the ARIC (Atherosclerosis Risk in Communities) study treated with SSRI antidepressants (mean age, 63 ± 10 years, followed for a median of 13.5 years) report a trend for increased risk of cardiovascular disease and stroke (hazard ratios, 1.10 and 1.07, respectively) as compared to patients treated with non-SSRI antidepressants (Almuwaqqat et al., 2019). Consistent with the aforementioned findings, previous studies demonstrate that in depressed monkeys treatment with SSRIs, such as sertraline, also significantly increases atherosclerosis in the carotids and the coronary circulation (Shively et al., 2015; Shively et al., 2017; Silverstein-Metzler et al., 2017). Importantly, recent studies demonstrate that in mouse models of atherosclerosis chronic treatment with fluoxetine also promotes atherogenesis by up-regulating vascular inflammation (Rami et al., 2018) (**Figure 1**). Interestingly, treatment with a different SSRI, escitalopram, was shown to reduce atherosclerotic changes in high-fat diet–fed rats (Unis et al., 2014). The mechanisms underlying these discordant findings are presently unclear. Fluoxetine treatment was demonstrated to induce endothelial activation and promote endothelial cell–leukocyte interaction (Herr et al., 2014). Citalopram was also suggested to promote vascular inflammation by increasing leukocyte binding to endothelial cells (Rami et al., 2018). In addition, SSRIs may also modulate leukocyte function by interfering with Ca^2+^ signaling mechanisms (Gobin et al., 2015).

Serotonin is released by activated platelets, contributing to enhanced platelet aggregation and vasoconstriction. Platelets play complex roles in atherogenesis and vessel occlusion upon plaque rupture (Fuster et al., 1990). Importantly, depressed patients often exhibit a prothrombotic phenotype, which may be modulated by treatment with SSRIs (Lopez-Vilchez et al., 2014). Treatment with SSRI affects platelet activation (de Abajo, 2011; Hoirisch-Clapauch et al., 2014; Lopez-Vilchez et al., 2017) and release of platelet-derived growth factors (Jha et al., 2017) that are important regulators of both atherogenic processes and vascular function. Interestingly, treatment with the SSRI paroxetine has been associated with induction of transient ischemic attack (Manos and Wechsler, 2004), which may be due, at least in part, to the effects of SSRIs on platelet function. Thus, SSRIs may also interfere with atherogenic processes, thrombus formation, and vascular occlusion by modulating platelet function.

Fluoxetine and other SSRIs may also impair function of leukocytes that are critical for organismal defenses against viral infections (Gobin et al., 2013). Importantly, there are studies extant suggesting that chronic infection with cytomegalovirus (CMV), which exhibits endothelial tropism, promotes atherogenesis. Age-related immunosenescence is thought to contribute to increased prevalence and severity of CMV infection in older adults. Further studies are needed to determine the causal link between SSRI treatment in geriatric patients, immunosenescence, CMV infection, and exacerbation of age-related vascular pathologies.

Antidepressants may also promote weight gain and metabolic disturbances, which may also affect risk of cardiovascular diseases.

## Potential Progeronic Effects of Fluoxetine

Molecular screening studies on the model organism *Caenorhabditis elegans* have identified a range of compounds, including several clinically approved antidepressants, which regulate lifespan and/or cellular resilience to oxidative stressors (Petrascheck et al., 2007; Petrascheck et al., 2009; Ye et al., 2014). Interestingly, several compounds that exert similar antidepressant effects in humans exhibit markedly different effects on cellular aging processes in *C. elegans*. While the structurally related antidepressants mianserin and mirtazapine extend lifespan and increase cellular oxidative stress resistance, fluoxetine exerts marked progeronic effects (Rangaraju et al., 2015). Despite recent advances in geroscience (Ashpole et al., 2017; Bennis et al., 2017; Fang et al., 2017; Fougere et al., 2017; Kim et al., 2017; Konopka et al., 2017; Podlutsky et al., 2017; Urfer et al., 2017) presently its unknown, which pathways involved in regulation of longevity and cellular resilience are modulated by SSRIs. The evolutionarily conserved mTOR (mechanistic target of rapamycin) pathway plays an integral role in the coordination of metabolism, protein synthesis, cell growth, and inflammation and thereby regulates cellular stress resistance, modulates aging processes, and determines mammalian lifespan (Harrison et al., 2009; Lin et al., 2013; Dai et al., 2014; Fok et al., 2014; Lesniewski et al., 2017). Inhibition of mTOR signaling by rapamycin treatment was shown to extend rodent lifespan (Harrison et al., 2009). Interestingly, there is initial evidence that fluoxetine activates mTOR (Liu et al., 2015). Further studies are warranted to determine how fluoxetine alters critical cellular mechanisms of aging in humans and to elucidate whether its action on mTOR may potentially exert progeronic effects. Studies testing the effects of fluoxetine treatment on a wide range of basic cellular mechanisms of aging as well as age-related changes in physiological functions (Beauchet et al., 2017; Belghali et al., 2017; Blodgett et al., 2017; Callisaya et al., 2017; Csiszar et al., 2017; Grimmig et al., 2017; Liu et al., 2017; Tarantini et al., 2017a; Tarantini et al., 2017b; Tarantini et al., 2017c; Ungvari et al., 2017) would be quite informative in that regard. Another evolutionarily conserved mechanism of aging involves dysregulation of IGF-1 signaling (Ashpole et al., 2017). Several studies have demonstrated that age-related decline in IGF-1 levels contributes significantly to brain and cerebromicrovascular aging (Mitschelen et al., 2011; Ungvari and Csiszar, 2012; Sonntag et al., 2013; Toth et al., 2014; Toth et al., 2015a; Tarantini et al., 2016; Tarantini et al., 2017c). Importantly, there is initial evidence that fluoxetine may affect IGF-1 levels in the central nervous system (Engesser-Cesar et al., 2007; Grunbaum-Novak et al., 2008), but the functional consequences of this effect are not well understood. Increased oxidative stress is a hallmark of aging, including vascular aging. Interestingly, chronic fluoxetine treatment was shown to induce increased oxidative stress in multiple organs (Zlatkovic et al., 2014) and aging-like phenotypic changes in vascular endothelial function in preclinical models (Simplicio et al., 2015).

## Cardiac Side Effects Of SSRIs In Aging

An increasing numbers of older patients with heart failure who develop depressive symptoms are treated with fluoxetine and other SSRIs. The cardiac side effects and toxicity of tricyclic antidepressants, manifested as slowing of intraventricular conduction, have been well documented in medical literature. Although fluoxetine causes significantly fewer cardiac side effects, there are reports in the literature on dysrhythmias, including atrial fibrillation or bradycardia and syncope associated with fluoxetine treatment and overdose (for an overview, see References Pacher et al., 1998; Pacher et al., 1999b; Pacher and Kecskemeti, 2004). Preclinical studies confirm that fluoxetine inhibits cardiac Na^+^ and Ca^2+^ channels, which may result in cardiac electrophysiological effects that are similar to those induced by tricyclic antidepressants (Pacher et al., 2000; Magyar et al., 2003). Experimental studies also suggest that chronic treatment with fluoxetine and other SSRIs may also compromise autonomic regulation of cardiac function in chronic heart failure (Henze et al., 2013). On the basis of the aforementioned observations, the age-related changes in the pharmacokinetics of SSRIs, and potential drug interactions in geriatric patients with cardiac disorders, Electrocardiogram (EKG) control may be suggested during SSRI therapy in the elderly (**Figure 1**).

## Perspectives

The diagnosis and treatment of depression in the elderly can be difficult, particularly in the presence of frailty and comorbidities. Factors to guide treatment choice can include the type of depression, previous response, and concurrent conditions (for strategies for selecting and switching antidepressants to treat geriatric patients, consult References Birrer and Vemuri, 2004; Frank, 2014). Selective serotonin reuptake inhibitor drugs, including fluoxetine, are the drug of choice for treatment of depression in the elderly: dosage adjustments are not required as frequently with SSRIs as with tricyclic antidepressants or monoamine oxidase inhibitors. Yet, despite the relative safety of SSRIs, these drugs also may have unwanted cardiovascular side effects. With advancing age, changes in drug metabolism and disposition may result in significant increases in the plasma concentrations of SSRIs, which may potentially promote, among others, cardiovascular and cerebrovascular pathologies. The prescriber should be alert to the potential adverse cardiovascular effects of SSRIs when treating depressed geriatric patients and consider the complex interaction between polypharmacy and potential cardiovascular morbidities. Psychological therapies should also be considered for geriatric patients with depression because of the increased vulnerability of these subjects to adverse effects associated with antidepressant treatment.

## Author Contributions

ZU, ST, AY, and AC provided substantial contributions to the conception or design of the work; or the acquisition, analysis or interpretation of data for the work; ZU, ST, AY, and AC contributed to the drafting the work or revising it critically for important intellectual content; ZU, ST, AY, and AC provided approval for publication of the content and agree to be accountable for all aspects of the work in ensuring that questions related to the accuracy or integrity of any part of the work are appropriately investigated and resolved.

## Funding

This work was supported by grants from the American Heart Association (ST), the Oklahoma Center for the Advancement of Science and Technology (to AC, AY, ZU), the National Institute on Aging (R01-AG055395, R01-AG047879), the National Institute of Neurological Disorders and Stroke (NINDS; R01-NS100782, R01-NS056218), the Oklahoma Shared Clinical and Translational Resources (OSCTR) program funded by the National Institute of General Medical Sciences (U54GM104938, to AY), the Presbyterian Health Foundation (to ZU, AC, AY). The authors acknowledge the support from the NIA-funded Geroscience Training Program in Oklahoma (T32AG052363).

## Conflict of Interest Statement

The authors declare that the research was conducted in the absence of any commercial or financial relationships that could be construed as a potential conflict of interest.
